# Optical-Amplifier-Compatible Long-Distance Secure Key Generation Based on Random Phase Fluctuations for WDM Systems

**DOI:** 10.3390/s20216296

**Published:** 2020-11-05

**Authors:** Ben Wu, Yue-Kai Huang

**Affiliations:** 1 Department of Electrical and Computer Engineering, Rowan University, 201 Mullica Hill Rd., Glassboro, NJ 08028, USA; 2 NEC Laboratories America, Inc., Princeton, NJ 08540, USA; kai@nec-labs.com

**Keywords:** fiber optics communications, optical security and encryption, phase fluctuations

## Abstract

We proposed and experimentally demonstrated a secure key generation and distribution system that is compatible with optical amplifiers and standard wavelength-division multiplexing (WDM) transmission systems. The key is generated from the phase fluctuations induced by environmental instabilities. The key generation system is tested in a 240 km bidirectional fiber-pair link with multiple optical amplifiers. To demonstrate the compatibility with WDM systems, 38 WDM channels are transmitted together with the key distribution channel. The secret key is protected against eavesdropping and coherence detection attack by the wide-band property of the signal carrier and the fast-changing rate of the phase fluctuations.

## 1. Introduction

Cryptography and data encryption are the fundamental elements of network security [1]. The effectiveness of the data encryption relies on the scheme to generate and distribute the key securely. If the key is attacked and exposed to an eavesdropper, the encrypted signal can be easily decrypted. Traditional key generation schemes are based on software algorithms. For example, the widely used Rivest–Shamir–Adleman (RSA) cryptosystem is based on the difficulty to factor the product of two large prime numbers [2]. The security of the system relies on computational resources available for the eavesdropper, while the increasing power of quantum computing raises a potential challenge to the software-based key generation schemes [3,4,5,6].

The physical layer encryption and key generation scheme provides an alternative solution to the network security [7,8,9]. One of the most widely studied physical layer key generation schemes is the quantum key distribution (QKD) [10,11,12]. The security of a QKD system relies on the fact that without knowing the eigenvalues of the quantum states, the eavesdropper cannot measure the signal, which is the quantum state of a single photon. Compared with traditional software-based key distribution methods, the physical layer key generation methods take advantages of the physical properties of the transmission media. If these physical properties are not properly recorded in real time, the data are lost permanently. The physical property for QKD is the quantum state of a single photon. If the eavesdropper does not know the eigenvalues of the quantum states, the original signals are lost at the time as the signals are being received by the eavesdropper. In this case, even the eavesdropper has unlimited computational power; the physical properties of the transmission media are not fully digitized for post-processing and signal recovery.

In this paper, we experimentally demonstrate a key generation and distribution system that is compatible with both optical amplifiers for long-range transmission and standard wavelength-division multiplexing (WDM) systems. The keys are generated from the random fluctuations of phase in fiber interferometers [13]. Environmental instabilities, including temperature changes and mechanical vibrations, induce random refractive index changes in fiber interferometers, which are deployed at each one of the communicating pair with long optical delays, and are then converted to random phase signals at both ends to generate digital keys. As a physical layer key generation scheme, the proposed system generates keys from phase randomness and if the analog phase signals are not recorded properly, the keys are lost and cannot be recovered.

The key generation system is demonstrated in a 240 km long fiber link with three optical amplifiers, and the key distribution channel cotransmits with 38 WDM communication channels. The proposed system is compatible with WDM channels and can be implemented over the existing fiber-optic networks based on three facts: (1) Standard optical fiber and fiber amplifiers are used to transmit signals for the key generation system. The key generation channel shares the C-band optical amplifiers with the other 38 WDM channels to compensate the attenuation from standard single mode fiber (SSMD). (2) The key generation system uses the bandpass filter with the same power extinction ratio as the standard WDM filters to download the key generation channel from the networks. (3) Since the power level of the key generation channel is comparable to the power level of the other WDM channels, photodetectors for standard WDM channels are directly used to receive signals of the key generation channel, and the key generation channel is installed with low cost. The proposed system can be implemented by plugging in a pair of key generators to the existing fiber networks for the two users (Alice and Bob) that require synchronized keys.

The security of the proposed system is based on the random phase fluctuations, and such phase fluctuations always exist in fiber interferometers. A pair of matched optical delay lengths is introduced to the two users that share keys. The matched optical delay lengths are in km range and coherence length of the broadband source is in mm range. If the eavesdropper tries to measure the phase fluctuations, another optical delay length has to be applied. The additional optical delay length, which is also in km range, introduces new phase fluctuations, so the eavesdropper cannot accurately measure the keys.

## 2. Experimental Setup

**Figure 1:**
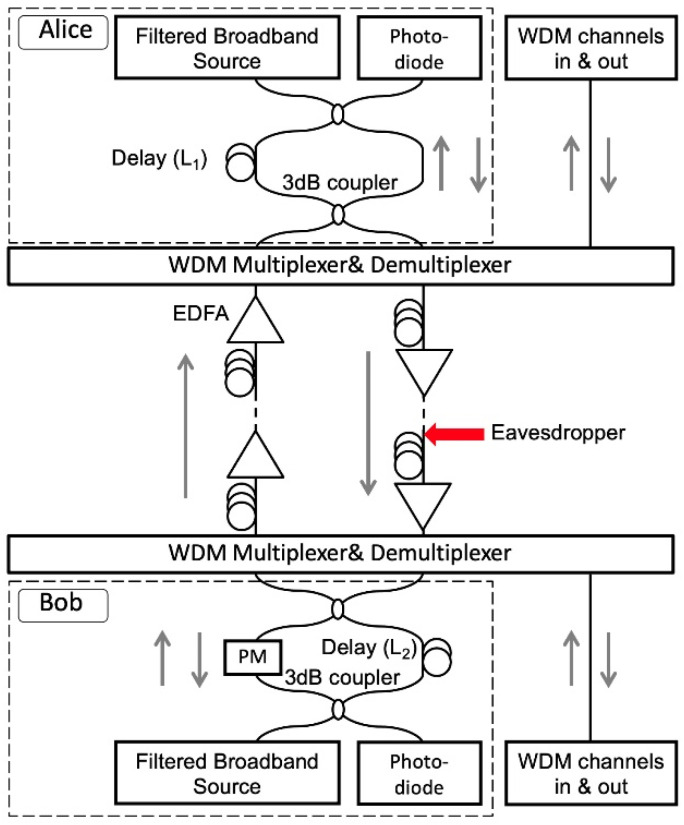
Experimental setup. (EDFA: erbium-doped fiber amplifier; WDM: wavelength-division multiplexing; PM: phase modulator).

Figure 1 shows the experimental setup of the key generation system, which consists of two Mach–Zehnder (MZ) delayed interferometers. The synchronized key signals are the results of the interference from interferometers at both ends. Both Alice and Bob send and receive signals with phases affected by the local temperature changes and mechanical vibrations. Since the signals in both directions go through the same path, the phase fluctuations and the interference results at the MZI outputs are the same at both ends, making key sharing possible. Broadband sources are used as the signal carriers to prevent the eavesdropper from measuring the phase directly with coherent detection. In the experiment, both Alice and Bob use a filtered amplified spontaneous emission (ASE) with bandwidth 150 GHz and center wavelength 1547.6 nm as the light source. Since the broadband source has short coherence length, which is 2 mm, the optical delays placed in Alice’s (L1) and Bob’s (L2) interferometers have to be matched within the 2 mm range to recover the phase information for key generation [14]. The optical delays used in the proposed system are typically in the range of tens of kilometers. Without knowing the delays in the interferometers, it is extremely difficult for the eavesdropper to scan the optical delays and find the coherence length in mm resolution. The delays (L1 and L2 in Figure 1) range from 20 m to 26 km in the experiment, which means the eavesdropper has to find a 2 mm range in up to a 26 km range in order to find the matching condition. More importantly, it should be noted that by building another fiber interferometer for eavesdropping, new phase randomness is created in addition to the transmitted phase information, thus reducing the accuracy of the eavesdropper’s measurement of the phase information.

The selection of 150 GHz bandwidth is a balanced solution that considers both the security of the system and the compatibility to WDM channels. The coherence length is inversely proportional to the bandwidth. Using the entire C-band for key generation enables the coherence length to be shorter than 2 mm, which creates a challenge for the eavesdropper to find the coherence length and the matching condition. However, it does not allow the existence of the other WDM channels. With 150 GHz bandwidth, the key generation channel can coexist with the other WDM channels in C-band. The selection of 26 km delay length not only provides large key space that challenges the eavesdropper to find the matching condition, but also generates fast-changing phase fluctuations that smear out the spectrum signatures that indicate the delay length. The experimental results for the spectrum signatures with different delay lengths are discussed in Section 3.2.

## 3. Results and Analysis

### 3.1. Key Generation without Phase Filter

**Figure 2:**
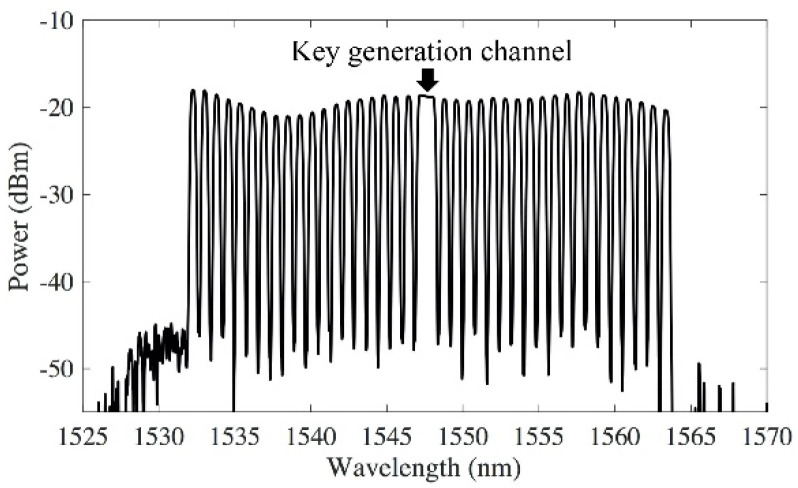
Spectrum of the key generation channel and the dummy WDM channels.

We demonstrated the transmission of the key distribution channel with 38 WDM channels using standard C-band transmission window. The key generation system is tested in both back-to-back transmission and a 240 km link. The 240 km bidirectional fiber-pair link includes three 80 km standard single mode fiber (SSMF) spans, each with erbium-doped fiber amplifier (EDFA) to compensate the average fiber loss of 17.5 dB. The 38 dummy WDM channels were emulated by carving another ASE source using a flexible-band wavelength selective switch (WSS) at 100 GHz spacing (Figure 2). The spectrum shows that the power spectral density (PDF) of the key distribution channel is comparable to the PDF of the other WDM channels, so the key generation channel can be downloaded from the network by using a bandpass filter with the same power extinction ratio as the standard WDM filters.

**Figure 3:**
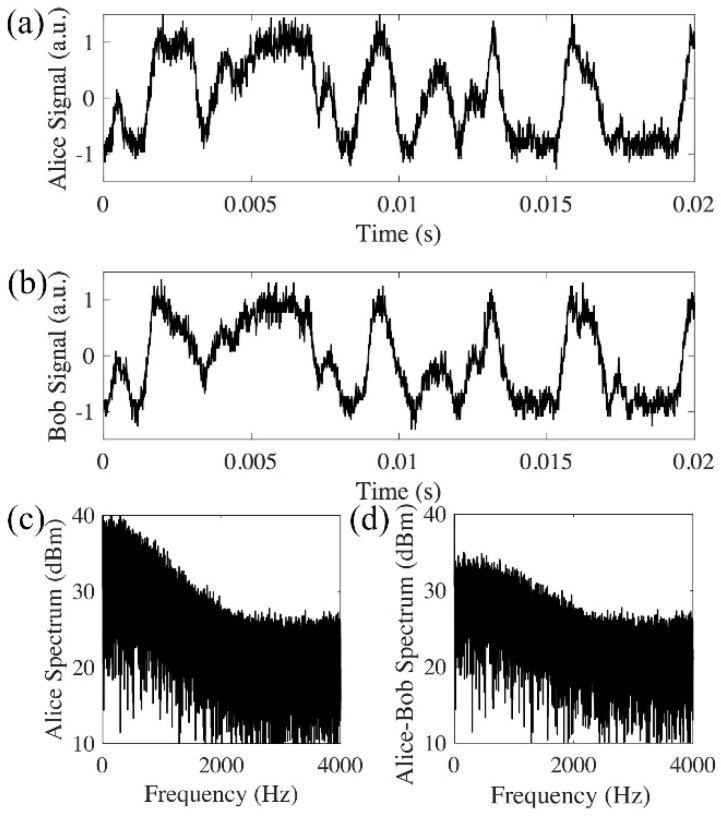
Direct detection of back-to-back signals with 26 km delays from both Alice and Bob. (**a**) Signal received by Alice; (**b**) signal received by Bob; (**c**) Alice’s spectrum; (**d**) differential spectrum between Alice and Bob has a much smaller amplitude than Alice’s spectrum (a.u.: arbitrary unit).

When 26 km optical delays are applied by both Alice’s and Bob’s MZIs, the two interference results by direct photo-detection match each other in back-to-back testing (Figure 3a,b). The phase randomness is generated from the 26 km optical delays deployed by both Alice and Bob. The spectrum analysis further proves the matched signal. Figure 3c is the spectrum of signal received by Alice, and shows that the signal exhibits frequency content up to 2 kHz. Figure 3d is the differential spectrum between the received signals, and is obtained by the Fourier transform of Alice’s signal minus Bob’s signal. The comparison between Alice’s spectrum (Figure 3c) and the differential spectrum (Figure 3d) shows that Alice’s spectrum is significantly larger than the differential spectrum up to 1 kHz, which further proves that Alice’s signal matches with Bob’s signal up to 1 kHz.

**Figure 4:**
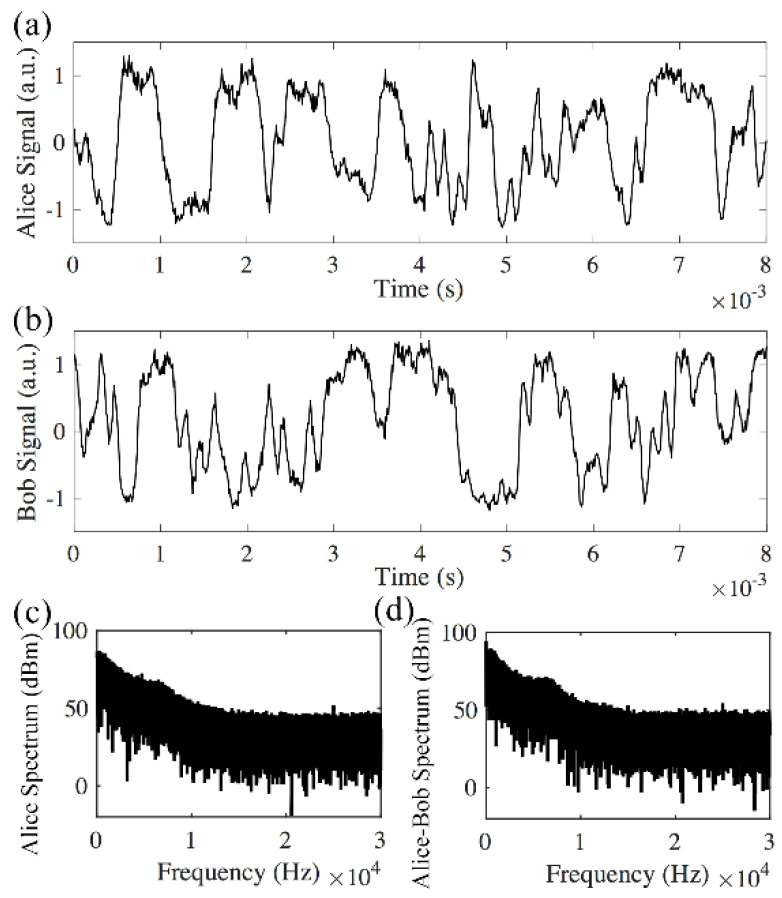
Direct detection of the signal after 240 km transmission with 26 km delays from both Alice and Bob. (**a**) Signal received by Alice; (**b**) signal received by Bob; (**c**) Alice’s spectrum; (**d**) differential spectrum between Alice and Bob has the same amplitude as Alice’s spectrum.

Figure 3 shows that Alice’s signal matches with Bob’s signal in the back-to-back transmission. After 240 km transmission, the interference results from Alice and Bob do not match (Figure 4a,b). This is because other than the rate of phase change induced by the environmental instability, the time of flight for the signal to travel between Alice and Bob also needs to be considered. In the experiment, the average time for the phase changes induced by the 26 km long MZI is in the order of 1 ms (Figure 3), while it takes the signal 1.2 ms to travel 240 km in the fiber. Thus, the signals in the two opposite directions will experience different phase changes. Figure 4 shows that the average phase changes induced by both the 26 km long delay of the MZI and the 240 km fiber are much faster than the phase changes in the back-to-back transmission system (Figure 3). The spectrum of the received signal has frequency contents above 10 kHz (Figure 4c). The comparison between spectrum received by one receiver (Alice in Figure 4c) and the differential spectrum (Figure 4d) shows whether the received signals are matched at different frequencies. Figure 3c, d shows that in a back-to-back link, the received signals are matched from direct current (DC) to 1 kHz, and in a logarithmic scale, most of the signal power lies within the range of DC to 1 kHz. In the 240 km link, Alice’s spectrum (Figure 4c) and the differential spectrum (Figure 4d) have the same amplitude from DC to 20 kHz, which means the received signal does not match in any frequency components.

There are two possible solutions to match the signals and synchronize the key: (1) The phase fluctuation speed can be reduced by using delay lengths less than 26 km, so the signals transmitted in both of the directions experience the same change. (2) A low-pass filter can be applied to remove the fast-changing components of the phase changes. Section 3.2 shows that the first solution undermines the security of the system. If the phase fluctuation speed is reduced, an eavesdropper is able to measure the delay length by the spectral pattern of the received signal. Section 3.3 demonstrates that with the second solution, the system generates matched keys in a secure way.

### 3.2. Security against Man-In-The-Middle Attack

The speed of phase fluctuations can be reduced by reducing the optical delay lengths in MZIs, since the changing speed of the phase is proportional to the optical delay length in the interferometer [15,16]. However, the slower phase changing speed poses a potential security weakness to the system. Eavesdroppers can use coherent detection to measure the location of the spectral fringes within the key signal spectrum. With the spectral fringes, it is possible to measure the optical delay lengths applied by Alice and Bob [17,18,19]. The challenge for an eavesdropper is that enough frequency resolution is required to observe the spectral fringes, and the frequency resolution is determined by the length of temporal data collected by the eavesdropper. With longer optical delay in MZ, a higher frequency resolution is required, and at the same time the fast phase changes will essentially smear out measurement results.

**Figure 5:**
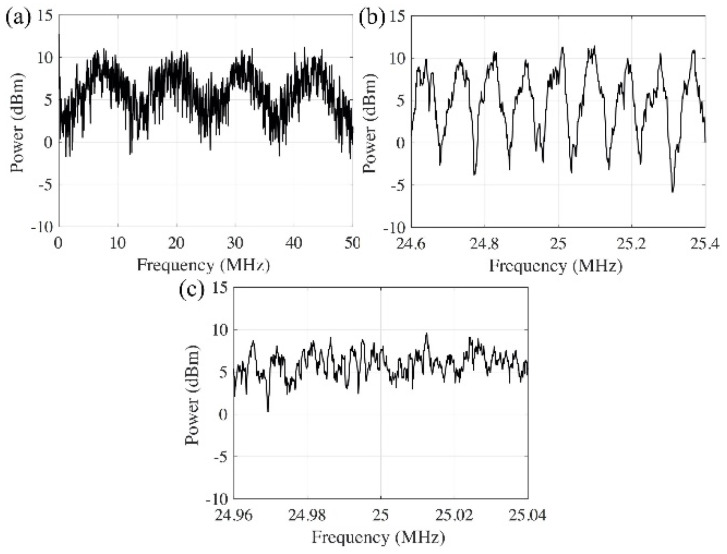
Spectrum of the signal from fiber interferometer with different optical delay length. (**a**) 20 m delay; (**b**) 2.3 km delay; (**c**) 26 km delay.

To determine the appropriate delay length that can defend the spectral-fringe-based attack, we emulate the eavesdropper (red arrow in Figure 1) and perform the attacks to the systems with different delay lengths. An ultra-stable fiber laser with 400 Hz linewidth is used as the local oscillator (LO) for the coherent detection. The Fourier transforms of the coherently detected signals show that when 20 m and 2.3 km delays are used (Figure 5a,b), the fringes in the spectrum can be clearly identified to track the phase fluctuation information within the interferometer. As for the 26 km delay, the fringes are smeared out by the fast-changing phase due to the long measurement time frame needed to achieve the frequency resolution required (Figure 5c). The experimental results show that the delay length of the fiber interferometer has to be longer than a few kilometers in order to protect against coherent detection attack.

### 3.3. Key Generation with Phase Filter

Since longer delay length is needed to protect the signal from eavesdropping, to match the interference results and enable the key exchange between Alice and Bob, the only solution is to remove the fast-changing component of the phase fluctuations so the slow-changing component of the phase fluctuations can be used for key generation. However, the low-pass filter cannot be directly applied on the interference results, which is the cosine term of the phase, because cosine is not a linear function for Fourier transform. Figure 4c, d compares the Fourier transforms of Alice’s direct detected signal and the differential spectrum between Alice’s and Bob’s. The results show that Alice’s spectrum has the same order of magnitude as the differential spectrum from DC to 10 kHz, meaning that the interference results do not match in either low- or high-frequency ranges.

**Figure 6:**
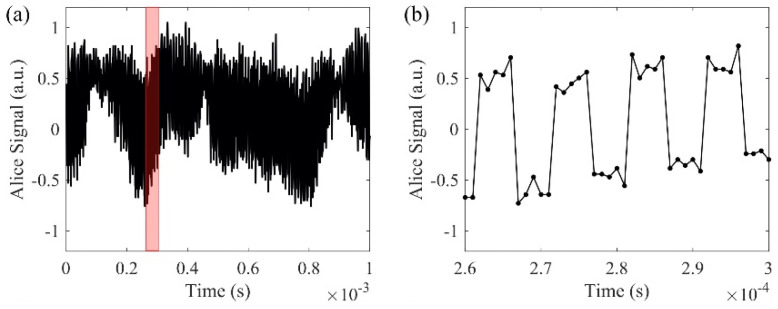
(**a**,**b**) are signals with phase modulator; (**b**) is the enlarged view of the red region in (**a**).

**Figure 7:**
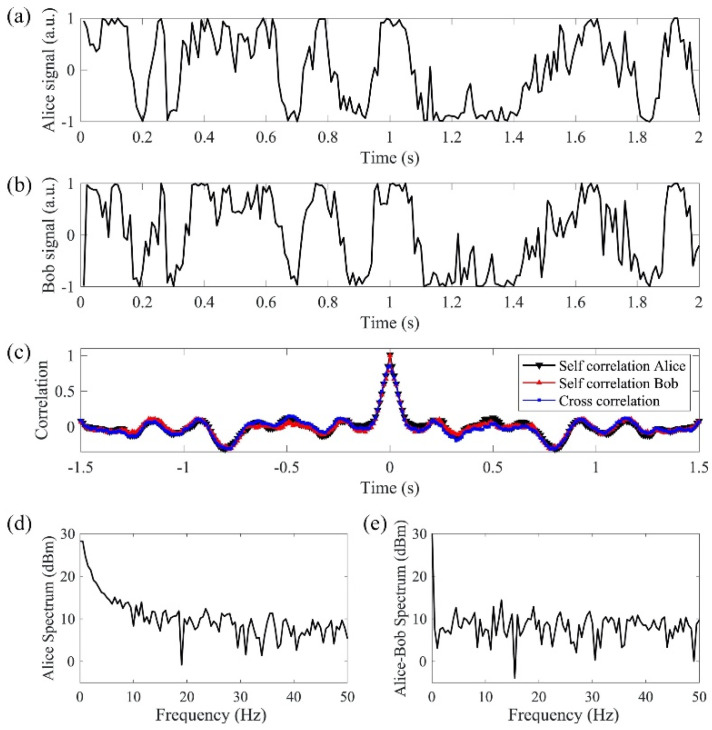
(**a**,**b**) are the received signals from Alice and Bob with high-frequency phase fluctuations filtered; (**c**) shows the self-correlations and cross-correlation of the signals; (**d**) Alice’s spectrum; (**e**) differential spectrum between Alice and Bob has a much smaller amplitude than Alice’s spectrum.

In order to detect the actual interference phase results, we use a phase modulator that switches between 0° and 90° at a rate of 100 kHz, which is more than 10 times faster than the phase changing rate (Figure 6a,b). Since the phase modulator switches at least one order of magnitude faster than the random phase signal, we assume that a neighboring 0° and 90° phase shift from the phase modulator is applied on the same random phase. In this case, both sine and cosine of the random phase are measured to obtain the random phase value. After digitization, a low-pass filter with 3 dB cutoff frequency at 50 Hz is applied to the measured phase signal digitally to remove the mismatched high-frequency phase components. Figure 7a, b shows the matched results from the cosine functions of the two filtered phase signals. The signals are transmitted in the 240 km bidirectional link. The self-correlation and cross-correlation of Alice’s and Bob’s filtered signals are plotted in Figure 7c. The self-correlations of Alice’s and Bob’s individual signals show the true temporal randomness of the generated key. The cross-correlation between Alice’s and Bob’s signals shows the matching performance of the key exchange with correlation peak of 0.8. The comparison between Alice’s spectrum (Figure 7d) and the differential spectrum between Alice and Bob (Figure 7e) shows that the differential spectrum has a much smaller amplitude than Alice’s spectrum. The differential spectrum further proves that with the 50 Hz low-pass filter applied on the phase, Alice’s signal matches with Bob’s signal.

To extract binary keys from the analog signals and reduce key errors, a buffer region is set in between the high and low levels, and the sampled points within the buffer region are dropped. The analog signals used to extract keys are sine and cosine functions of the recovered and filtered phases, which range from −1 to 1. The boundary of the buffer region is −v_o_ and v_o_ (0 < v_o_ < 1). If the analog signal is higher than the buffer region, a bit 1 is generated as the key. If the analog signal is lower than the buffer region, a bit 0 is generated as the key. The buffer region is optimized to minimize the dropped samples and key error rate (Table 1). Both sine and cosine functions of the measured phase are used to generate binary keys. The key generation achieves a rate of 90 bps and an error rate of 5 × 10^−3^, which can be corrected by hard decision FEC technique [20,21] and key reconciliation [22]. To ensure true randomness of the generated key, the 90 bps is further reduced to 20 bps based on the width of the correlation peak in Figure 7c. The width of correlation peak is 100 ms, which means two bits with intervals equal to or larger than 100 ms are not correlated. By using the cosine of the phase, a bit rate of 10 bps is obtained. Since sine and cosine functions are orthogonal, another 10 bps is obtained with the sine function.

By using standard optical amplifiers in WDM networks, the transmission distance can go beyond 240 km. The maximum transmission distance depends on two factors: (1) Key rate: To remove the unsynchronized phase signal, the 3 dB cutoff frequency of the low-pass phase filter for the 240 km link is 50 Hz. With the transmission distance longer than 240 km, the cutoff frequency is inversely proportional to the transmission distance. (2) Signal-to-noise ratio (SNR): The signal carrier of the key generation channel is wide-band ASE noise. The SNR of the key distribution channel is different from the SNR of a standard communication channel that uses a laser as the signal carrier [23]. For the standard communication channel, the ASE noise only exists in the denominator of the SNR. For the key distribution channel, the power spectral density of the ASE noise affects both the numerator and denominator of the SNR, which means the key distribution channel has shorter maximum transmission distance than the standard communication channel. The theorical model and experimental results of the maximum transmission distance for a communication channel that uses ASE noise as the signal carrier is analyzed in [23].

### 3.4. Analysis of Information Leakage

Section 3.2 has demonstrated that if the optical delay is longer than a few kilometers, the eavesdropper is not able to measure the spectral fringes and thus cannot measure the optical delay by coherence detection. This section shows that the system is protected from the information leakage, and even if the eavesdropper knows the right optical delay length, he or she cannot measure the phase information from Alice and Bob. The analysis in this section is based on the assumption that the eavesdropper uses brute force attack to scan the 26 km fiber with high resolution and finds the 2 mm coherence length. Under such assumption, the eavesdropper uses the same optical delay as the authorized users to measure the phase information from Alice (*φ_A_*) or Bob (*φ_B_*). The 26 km optical delay applied by the eavesdropper generates additional phase randomness (*φ_E_*). Although the eavesdropper can use a feedback system to control the phase randomness, the feedback system applies to the sum of the phase change *φ_A_ + φ_E_*, which means the eavesdropper can only measure *φ_A_ + φ_E_* or *φ_B_ + φ_E_* and cannot accurately measure either *φ_A_* or *φ_B_*.

### 3.5. Description of the Protocol

In this section, the key distribution protocol is summarized step by step based on the experimental setup and results. The protocol includes the following five steps: (1) Alice and Bob match optical delays with preshared length. Based on the secure analysis in Section 3.2, the matched delay lengths range from a few kilometers to less than 100 km. The accuracy of the matched delay lengths is within 2 mm, which is the coherence length of the light source. (2) The delay lengths generate random phase differences between two light paths at the interferometers on both Alice’s (*φ*_A_ = *φ*_A1_ − *φ*_A2_) and Bob’s (*φ*_B_ = *φ*_B1_ − *φ*_B2_) sides, where φ represents phase, A and B represent Alice and Bob, respectively, and 1 and 2 represent two paths in the fiber interferometers. In both of the transmission directions, from Alice to Bob and from Bob to Alice, the signal carriers experience the same phase change (*φ*_A1_ − *φ*_A2_ +*φ*_B1_ − *φ*_B2_), which generates synchronized keys. (3) The phase changes *φ*_A1_ − *φ*_A2_ and *φ*_B1_ − *φ*_B2_ are time-dependent, and have frequency components from DC to 10 kHz (Figure 4). It takes 1.2 ms for the signal carriers to transmit through the 240 km fiber link, and if either *φ*_A1_ − *φ*_A2_ or *φ*_B1_ − *φ*_B2_ changes during the 1.2 ms, Alice and Bob receive mismatched signals. Therefore, low-pass digital filters are used at both Alice’s and Bob’s sides to remove the fast-changing frequency components of the phase changes *φ*_A1_ − *φ*_A2_ and *φ*_B1_ − *φ*_B2_., and only the slow-changing frequency components are used to generate the keys. (4) A phase modulator that switches between 0° and 90° is applied at the receiver (Figure 1), so both sine and cosine of the phase changes are received, and the phase information can be recovered. (5) Alice and Bob both generate binary digital keys based on the synchronized analog signals. A buffer region is set in between the high and low levels. Sampled signals above and below the buffer region generate digital keys of 1 and 0, and the sampled signals within the buffer region are dropped.

## 4. Conclusions

We propose and experimentally demonstrate a key distribution system that is compatible with optical amplifiers for long-distance communication and standard WDM systems. The keys are generated and digitized from the random phase fluctuations caused by environmental instabilities in fiber interferometers. A bidirectional transmission link with multiple EDFAs and 240 km length of optical fiber is demonstrated. The signal carrier of the key distribution channel is wide-band ASE noise from EDFA and shares C-band transmission spectrum with 38 neighboring WDM channels. The wide-band ASE noise and the fast-changing phase protect the optical delay in the key distribution system from being measured by an eavesdropper with coherent detection attack.

## Figures and Tables

**Table 1:** Ratios of dropped/accepted sample and key error.

Description	Percentage
Dropped samples	54%
Accepted samples	46%
Key error	0.5%
Correct key	99.5%
